# Clinically Inspired Skin Lesion Classification through the Detection of Dermoscopic Criteria for Basal Cell Carcinoma

**DOI:** 10.3390/jimaging8070197

**Published:** 2022-07-12

**Authors:** Carmen Serrano, Manuel Lazo, Amalia Serrano, Tomás Toledo-Pastrana, Rubén Barros-Tornay, Begoña Acha

**Affiliations:** 1 Dpto. Teoría de la Señal y Comunicaciones, Universidad de Sevilla, Camino de los Descubrimientos s/n, 41092 Seville, Spain; manu11235@gmail.com (M.L.); bacha@us.es (B.A.); 2 Hospital Universitario Virgen Macarena, Calle Dr. Fedriani, 3, 41009 Seville, Spain; amaliaserranog@gmail.com; 3 Hospitales Quironsalud Infanta Luisa y Sagrado Corazón, Calle San Jacinto, 87, 41010 Seville, Spain; ttoledop@gmail.com; 4 Hospital Universitario de Cabueñes, Los Prados, 395, 33394 Gijón, Spain; rubbartor@gmail.com

**Keywords:** basal cell carcinoma, color cooccurrence matrix, deep learning, color appearance models, clinically inspired classification, dermatology

## Abstract

*Background and Objective*. Skin cancer is the most common cancer worldwide. One of the most common non-melanoma tumors is basal cell carcinoma (BCC), which accounts for 75% of all skin cancers. There are many benign lesions that can be confused with these types of cancers, leading to unnecessary biopsies. In this paper, a new method to identify the different BCC dermoscopic patterns present in a skin lesion is presented. In addition, this information is applied to classify skin lesions into BCC and non-BCC. *Methods*. The proposed method combines the information provided by the original dermoscopic image, introduced in a convolutional neural network (CNN), with deep and handcrafted features extracted from color and texture analysis of the image. This color analysis is performed by transforming the image into a uniform color space and into a color appearance model. To demonstrate the validity of the method, a comparison between the classification obtained employing exclusively a CNN with the original image as input and the classification with additional color and texture features is presented. Furthermore, an exhaustive comparison of classification employing different color and texture measures derived from different color spaces is presented. *Results*. Results show that the classifier with additional color and texture features outperforms a CNN whose input is only the original image. Another important achievement is that a new color cooccurrence matrix, proposed in this paper, improves the results obtained with other texture measures. Finally, sensitivity of 0.99, specificity of 0.94 and accuracy of 0.97 are achieved when lesions are classified into BCC or non-BCC. *Conclusions*. To the best of our knowledge, this is the first time that a methodology to detect all the possible patterns that can be present in a BCC lesion is proposed. This detection leads to a clinically explainable classification into BCC and non-BCC lesions. In this sense, the classification of the proposed tool is based on the detection of the dermoscopic features that dermatologists employ for their diagnosis.

## 1. Introduction

Skin cancer is the most common cancer worldwide [1]. There are two main types of skin cancer: melanoma and non-melanoma. The most common non-melanoma tumors are basal cell carcinoma (BCC) and squamous cell carcinoma (SCC). BCC accounts for 75% of all skin cancers and it is the most common malignant tumor in white populations [2]. The detection of this cancer is executed by visual inspection by a skilled dermatologist, but there are many benign lesions that can be confused with these types of cancers, leading to unnecessary biopsies in a proportion of five biopsies versus one actual cancer case [3].

### 1.1. Related Work in the Literature

Ruffano et al. published a comparative study of the use of 24 computer-aided diagnosis (CAD) tools for skin cancer detection and they concluded that CAD systems obtained high sensitivity and could be used as a back-up for specialist diagnosis in a carefully selected patient population, but there is no evidence that, for daily clinical practice, they will be useful to assist clinicians. In this sense, prospective comparative studies are required [4]. Marka et al. present a review of techniques focused on the automatic detection of non-melanoma skin cancer [5]. Their conclusion is that the overall quality of evidence for the diagnostic accuracy of the automatic classification of non-melanoma skin cancer is moderate. The most common limitations of the studies included in the review are the overlapping training and test sets, the non-biopsy-proven references and the non-consecutive samples.

Lately, the use of artificial intelligence (AI) using deep neural networks in the classification of images or object recognition has increased. Specifically, convolutional neural networks (CNN) have become a powerful classification tool. The large databases available to the research community and the improvements in graphic processing unit (GPU) capabilities have contributed to this success. Despite all the above, there is still a wide improvement margin in developing CAD tools in order to be reliable. Most of the AI works published in the field of skin cancer detection have been focused on melanoma [6,7,8,9]. On the other hand, works devoted to detecting non-melanoma skin cancer are uncommon in the literature. Usually, these papers classify skin lesions into different classes of diseases [10,11,12,13,14,15].

The main concern about CAD tools based on deep learning (DL) is the lack of explainability of the classification result. Deep neural networks are considered as black boxes, which give a label for each class, but without revealing the internal decisions that have been taken to reach this label. Lately, different efforts have been directed towards the development of CAD tools for a clinical explainable classification of skin cancer [16,17,18,19] In this regard, in this paper, a CAD tool that provides an explainable detection of BCC is presented.

There are different types of images that physicians use to diagnose the skin lesion (spectroscopy [20], optical coherence tomography, thermography, multispectral images, wide field images [21], ultrasonography [22,23], etc.), but the most used and the simplest one is the digital dermoscopy—that is, digital color photography enhanced by a dermoscope [24].

Dermatologists diagnose BCC from dermoscopic images by detecting different high-level features or dermoscopic criteria. BCC has the most clearly defined clinical criteria [2,25]. Dermoscopic criteria for BCC are branching and linear vessels (arborising and superficial telangiectasia), multiple erosions, ulceration, bluish-gray clods of variable size (ovoid nests, globules and focused dots), radial lines connected to a common base (leaf-like areas), radial lines converging to a central dot or clod (spoke-wheel areas), clods within a clod (concentric structure) and absence of brown reticular lines (pigment network) [2,26].

In the last few years, some works have been focused on the detection of BCC in dermoscopic images. However, to the best of our knowledge, there are a very limited number of works devoted to detecting some of the dermoscopic features that dermatologists employ to diagnose BCC, and none of these works focus on the detection of all the BCC dermoscopic features. Specifically, Cheng et al. detected telangiectasia [27]. Kharazmi et al. analyzed vascular structures to classify dermoscopic images into BCC or non-BCC [28,29]. Guvenc et al. [30] put their effort into detecting blue-gray ovoids, furthering the goal of automatic BCC detection. They extracted 24 color features and concluded that color features allow accurate localization of these structures. Cheng et al. used different types of features (patient age, gender, lesion size and location, dermoscopic patterns) to classify BCC vs. benign lesions with a neural network [31] Kefel et al. extracted measures of smoothness and brightness in red, green, blue and luminance bands to detect semitranslucent and pink blush areas in BCCs [32]. One of the problems encountered while trying to solve this issue is the lack of databases with annotated data (weakly annotated or pixelwise annotated) regarding the dermoscopic criteria found in BCC.

Color is a very important feature to be considered in order to detect and classify images of skin lesions [33,34,35,36,37]. In the particular case of detecting BCC dermoscopic criteria, the color information of the image is crucial. To the best of our knowledge, papers found in the literature that apply DL techniques to classify BCC do not analyze the color information of the image, but they use *RGB* BCC images without any color processing as inputs to a CNN. None of the papers found in the literature include a study of the influence of color on the classification results.

Lessons learned from this literature review are summarized below:

- There are works in the literature that detect one or several BCC patterns, but none of them detect all the BCC patterns that dermatologists employ to diagnose;
- There are works in the literature that employ deep neural networks (DNN) to classify BCC versus other dermatological lesions, but attempts to ensure the clinical explainability of the classification are limited;
- Color analysis is crucial to analyze pigmented lesions;
- Existing CAD tools for skin cancer detection lack a prospective study that validates their results.

The authors have worked for more than two decades in skin lesion analysis. Specifically, most of their publications have been focused on burn images and dermoscopic images. They have developed computer-aided diagnosis (CAD) tools for applications focusing on the detection of both color and texture features [38,39,40,41].

Color and texture analysis of this type of image has been crucial in order to obtain good classification and segmentation results. The authors have developed many different methods that calculate new color and texture features [34,42,43].

Lately, they have also applied methods based on DL methodology [44,45], but they came to the conclusion that the classification results attained with these methods are not easy to explain and the optimum architecture to obtain the classification results is not easy to determine. This is why, in the proposed method, together with the classification into BCC or non-BCC, we provide an explanation of this classification.

In this paper, we apply the findings of color science to the detection of BCC dermoscopic features and the classification of the lesion into BCC and non-BCC. The intended use of the proposed method is as a prioritization tool to evaluate all the images received at the hospital by teledermatology, which have recently seen a significant increase due to the high incidence of skin cancer, resulting in an overload for dermatologists.

### 1.2. Our Contributions

The main contributions of the paper to the state-of-the-art can be summarized as follows:

(1) Detection of all BCC dermoscopic features.

To the best of our knowledge, no other works in the literature have developed a methodology to detect all the possible patterns that can be present in a BCC lesion.

(2) Clinically inspired classification of lesions into BCC/non BCC.

To the best of our knowledge, no other works in the literature have attained this goal. The detection of BCC dermoscopic patterns has been used in the clinically inspired classification of a lesion into BCC/non BCC. Physicians prefer to identify the patterns present in the lesion, instead of only the binary classification into BCC/non BCC. That is, they need an explainable diagnosis. Most of the works in the literature perform a binary classification.

There are some works in the literature that, in other applications, have tried to explain the classification by identifying the main areas of activation of the DNN. Differently, in our approach, we try to explain the classification based on the clinical signs found in the image.

(3) For BCC dermoscopic feature classification, we propose to combine color and texture analysis.

New parameters extracted from a new, perceptually inspired color cooccurrence matrix have been designed. To the best of our knowledge, no other works in the literature have considered uniform color spaces and recent advances in color appearance models for the computation of texture parameters extracted from a cooccurrence matrix.

(4) An annotated database of the main BCC dermoscopic features has been developed. A software application, so that dermatologists can annotate the images, has been designed.

There are no public annotated databases of all the BCC dermoscopic criteria. In this project, a new database composed of 692 BCC images and 2221 different patterns has been collected.

## 2. Methods

### 2.1. Design Considerations

The first design consideration is related to the development of the annotated database. Due to the high care load, physicians have limited time to annotate the database. Thus, an annotation tool with a user-friendly interface has been designed, where physicians select, with a simple mouse click, the pattern that they find in the lesion. This tool has been installed in the computer room of the Dermatology Unit at Hospital Universitario Virgen Macarena, Seville (Spain).

Due to the small database, a DNN trained exclusively with the BCC pattern images did not attain good results. Thus, it was decided to add color and texture features to improve the performance.

On the other hand, a conventional machine learning tool based only on color and texture features was also tested. However, a DL approach that combined image and color and texture information improved the classification results.

The drawbacks of DL are the high computational cost and the requirement of a large database. The first problem was overcome with a computer equipped with a high-performance GPU. The second problem was overcome with transfer learning and by adding color and texture handcrafted features, as explained above.

### 2.2. Database

**Figure 1:**
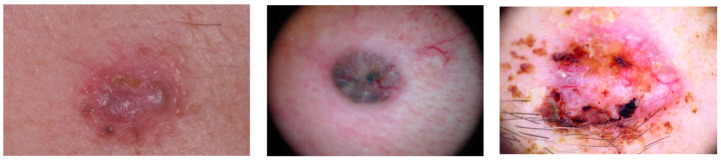
ISIC BCC image samples (Challenge 2018).

The first problem encountered is the limited public databases with BCC images. In the challenges of 2018 and 2019 organized by the International Skin Imaging Collaboration (ISIC) project, a set of 514 and 3323 BCC dermoscopic images was made available, respectively [46,47,48,49]. In Figure 1, three examples of BCC images from ISIC Challenge 2018 are shown. There are no public databases with the segmentation of the different patterns that can be found inside a BCC lesion.

In order to collect a database composed of images representing BCC dermoscopic structures, we have developed a user-friendly program that has been installed on computers located at the Hospital Universitario Virgen Macarena, Seville, Spain. Using this software, the experts have been able to manually segment 256 × 256 pixel images, where one or more patterns are presented. As this is a tedious task, such a software tool can be helpful and time-saving. Images were collected during 9 months approximately. All the BCC images used in the evaluation have been excised and biopsied. The approximate time interval between the dermatoscopic image acquisition and the dermatologist’s diagnosis was less than 10 days. The biopsy was performed less than 90 days from the image acquisition. The eligibility criterion for selecting BCC cases was patients with a BCC diagnosis in teledermatology consultation, and subsequently confirmed by biopsy. Patients belonged to the Andalusian Health System and attended medical centers assigned to the area of influence of Hospital Universitario Virgen Macarena, Seville (Spain). The inclusion criterion was an age over 18 years (non-pediatric).

All the lesions that were not BCC were extracted from the HAM10000 database, where more than 50% of lesions have been confirmed by pathology, while the ground truth for the rest of the cases was either follow-up, expert consensus or confirmation by in vivo confocal microscopy [49]. Most non-BCC lesions were pigmented benign keratosis, but other non-BCC lesions have been chosen as well to build the non-BCC database (nevus, lentigo, seborrheic keratosis, actinic keratosis, melanoma, squamous cell carcinoma and hemangioma).

**Figure 2:**
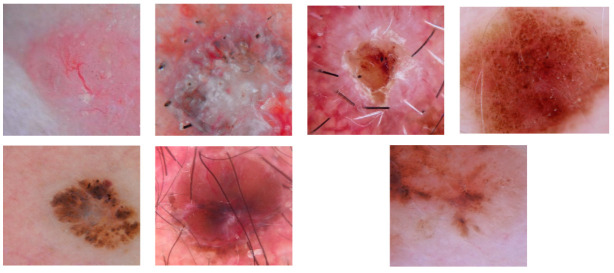
Top row, from left to right: telangiectasia, multiple B/G globules, ulceration and pigment network; bottom row: spoke-wheel, blue-gray ovoids and maple leaf.

In Figure 2, examples of the different patterns that can be found in BCC lesions extracted with the developed software tool are shown. All of them are positive criteria, except for the pigment network, which represents a negative criterion. In Table 1, the number of images for each pattern in the dataset is presented. In total, in the database, there are 692 BCCs and 671 non-BCC images.

In order to overcome the limitations imposed by our small database, data augmentation was performed. The number of images in the training database has been multiplied by 4 (from 1371 images to 5484). The transformation applied to each image consisted of 90°, 180° and 270° rotations and random horizontal flipping and vertical flipping.

As stated by Barata et al. [16], the features extracted by CNNs are color-sensitive, confirming the findings of Mahbod [50], who showed that color normalization has a positive impact on the performance of a CNN. However, in our case, as the images have been taken under the same conditions, color normalization has not been necessary.

We have considered that performing a color transformation for data augmentation should not be applied when dealing with medical images whose diagnosis is based on color.

### 2.3. Color Processing

In this paper, a color analysis is applied in order to test how color influences the classification of BCC lesions. For this purpose, an analysis of the main colors present in a lesion is performed. Different color spaces and color distance metrics are tested. 

#### 2.3.1. Uniform Color Spaces and Perceptual Color Differences

Uniform color spaces are color systems where Euclidean distances correlate well with perceived color differences. In 1976, the Commission Internationale de l’Eclairage (CIE) standardized two color spaces, L*u*v* and L*a*b*, with the aim of providing a tool to measure color differences perceived by human observers [51].

The mathematical definition of the CIELAB color difference between two colors, with color coordinates $\left(L_{1}^{*},\;a_{1}^{*},\;b_{1}^{*}\right)$ and $\left(L_{2}^{*},\;a_{2}^{*},\;b_{2}^{*}\right)$, is

(1) $$\Delta E_{ab}^{*}={\left[{\left(L_{2}^{*}-L_{1}^{*}\right)}^{2}+{\left(a_{2}^{*}-a_{1}^{*}\right)}^{2}+{\left(b_{2}^{*}-b_{1}^{*}\right)}^{2}\right]}^{1/2}\;=\;{\left[\Delta L^{*2}+\Delta a^{*2}+\Delta b^{*2}\right]}^{1/2}$$

The unsatisfactory uniformity of the CIE L*a*b* space prompted researchers to investigate better color difference formulas and new color systems. 

CIE color systems can accurately predict whether two colors will match for an average observer, but cannot provide information about the color appearance of these stimuli. Colors, usually, do not appear isolated in a scene, and the color appearance is strongly affected by the viewing conditions and the surroundings of color stimuli [52]. In this work, the color appearance of the different regions present in a lesion is targeted for analysis. Thus, a color appearance model is applied in this analysis. Specifically, the recent CIECAM16 color appearance model, recommended by CIE to replace CIECAM02, is used [53].

CIECAM16 needs as inputs the *X* and *Y* coordinates of the stimuli from *XYZ* color space, the color coordinates of the illuminant and parameters about the viewing surroundings and background. Then, it performs an illuminant and color adaptation and it obtains the following color appearance correlates: lightness *J*, chroma *C*, hue composition *H*, hue angle *h*, colorfulness *M*, saturation *s* and brightness *Q* [53]. Following the color appearance terminology, *brightness* is the attribute of a visual sensation according to which an area appears to emit more or less light, and *lightness* is the brightness of an area judged relative to the brightness of a similarly illuminated area that appears to be white or highly transmitting. *Colorfulness* is the attribute of a visual sensation according to which the perceived color of an area appears to be more or less chromatic; *chroma* is the colorfulness of an area, judged as a proportion of the brightness of a similarly illuminated area that appears white or highly transmitting; and *saturation* is the colorfulness of an area judged in proportion to its brightness. Hue is the attribute of a visual sensation according to which an area appears to be similar to one of the perceived colors—red, yellow, green and blue—or to a combination of two of them. *Hue composition* describes the perceived hue in terms of the percentages of two of the unique hues, whereas the *hue angle* describes the perceived hue as a quantity between 0° and 360° [52].

From this color appearance model, a uniform color space, CAM16-UCS, can be defined.

(2) $$J^{\prime }=\frac{1.7J}{1+0.007J}$$

(3) $$M^{\prime }=\ln\frac{1+0.0228M}{0.0228}$$

(4) $$a^{\prime }=M^{\prime }\cos\left(h\right)$$

(5) $$b¡=M^{\prime }\sin\left(h\right)$$

The color difference between two samples can be computed as the Euclidean distance between them in CAM16-UCS,

(6) $${\Delta E}^{\prime }=\;{\left[{{\Delta J}^{\prime }}^{2}+{{\Delta a}^{\prime }}^{2}+{{\Delta J}^{\prime }}^{2}+{{\Delta b}^{\prime }}^{2}\right]}^{1/2}$$

For more information about the CIECAM16 color appearance model, please refer to Appendix A of Li et al. [53].

#### 2.3.2. Perceptual Clustering and Relevant Color Identification

In order to improve and facilitate the learning process of the DNN classifier, we propose to introduce, as additional information, the main colors present in the lesion. Due to the small database, we consider that the training process might be facilitated through the introduction of the significant colors of the dermoscopic patterns.

Quantization of the colors of the images involves the election of a clustering algorithm, a color space and a distance metric. The use of Euclidean distances in uniform color spaces provides a perceptual clustering—that is, colors that are perceived as similar by a human observer will be assigned to the same cluster.

The K-means algorithm is the most popular partitional clustering algorithm [54]. When the distance metric in the partitioning space is Euclidean, cluster centers coincide with geometric centers (centroids). Thus, the updating of the centroids is trivial. However, when using non-Euclidean distance formulae, the updating of cluster centers requires specific algorithms to find them. As the color spaces utilized in this paper are perceptually uniform, color clustering employing the Euclidean distance is the most adequate to perform a perceptual clustering.

Identification of the main colors present in each pattern is performed in two steps:

(1) In an initial step, a color clustering is performed for all the training images for each BCC dermoscopic pattern. The cluster centers are initialized randomly. After this step, 18 color centroids for each pattern are obtained—that is, a total of 126 color centroids are determined.
(2) Many color centroids from different patterns are very similar. For this reason, in a second step, two centroids are merged if their distance is below a threshold. This threshold is automatically adjusted so that 20 color centroids are retained at the end.

**Figure 3:**
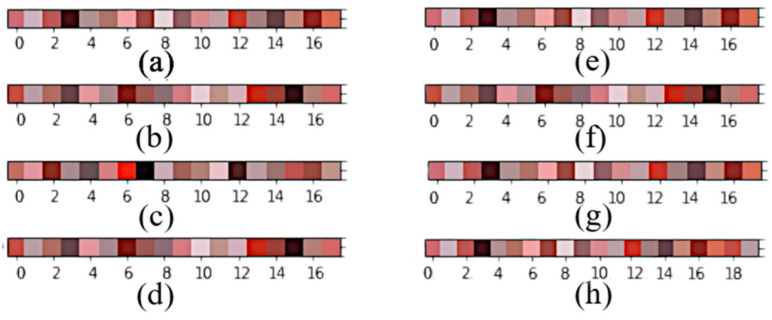
Representative colors of each BCC dermoscopic pattern in *RGB* color space. (**a**) Pigment network, (**b**) ulceration, (**c**) blue-gray ovoids, (**d**) multiple B/G globules, (**e**) maple leaf, (**f**) spoke-wheel, (**g**) telangiectasia, (**h**) the 20 final color centroids selected.

Figure 3 shows the most representative colors of each BCC dermoscopic pattern in *RGB* color space.

**Figure 4:**
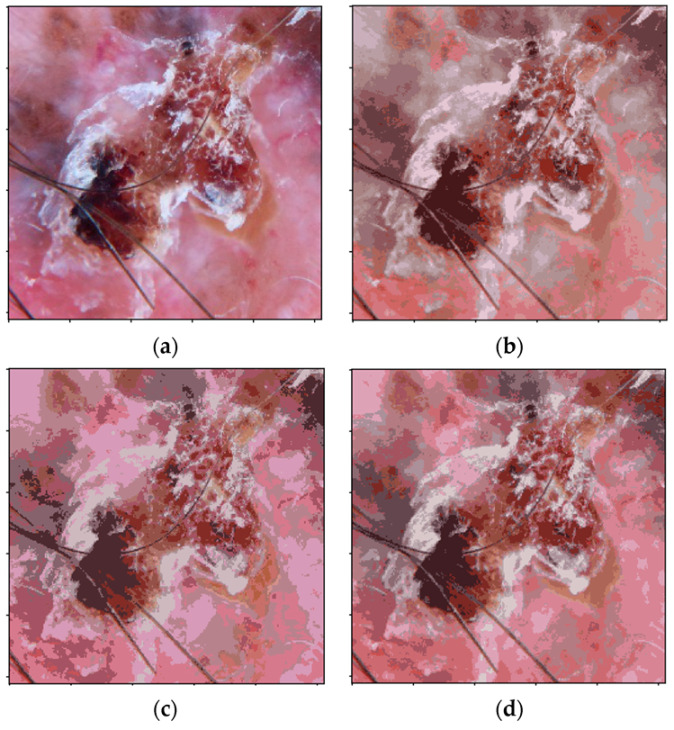
Color quantization in different color spaces. (**a**) Original image. (**b**) Image quantized in *RGB* color space (20 colors). (**c**) Image quantized in L*a*b* color space (18 colors). (**d**) Image quantized in CIECAM16-UCS (18 colors).

In Figure 4, an example of the quantization into the main BCC representative colors is presented. As can be observed in Figure 4, the pink color detected in the *RGB* color quantization does not match exactly with the pink color in the original image. Some pink regions in the original image are assigned to a gray centroid in the *RGB* quantization. These problems are observed in the two uniform color spaces, CIECAM16 and CIELAB. Both of them preserve well the color appearance of the image.

### 2.4. Texture Analysis

In the analysis of BCC patterns, texture plays an important role. Thus, the introduction of texture features as additional information in the neural network could help in the classification.

The gray-level cooccurrence matrix (GLCM) is a very useful tool in texture analysis, because it is based on an estimation of second-order gray-level statistics [55] (A GLCM defines the frequency of the cooccurrence of two gray levels at a given relative location in an image. Specifically, defining a gray level i, in a particular position, and gray level j, at a particular spatial relationship with the first one, GLCM provides an estimate, $P_{ij}$, of the probability of having pixel values i and j in these relative positions. However, some authors [56,57] have demonstrated that the introduction of color information facilitates the classification of color texture.

In this paper, two different methods to combine color and texture have been investigated:

(1) GLCM applied to the L* channel in the uniform color space L*a*b* along with color information, introduced to the network via the image quantized into the main colors. The L* channel represents the perceived relative brightness, and, thus, spatial distribution information can be captured with GLCM in L*.
(2) A new color cooccurrence matrix (CCM) applied to the color-quantized images according to color appearance information. The cooccurrence of the main colors present in the image is analyzed. As 20 main colors are detected, a matrix of 20 × 20 is obtained. For each element in the matrix, the probability of cooccurrence of color index i  and color index j, $P_{ij}$ in a particular pair of relative spatial positions, is estimated. In this case, when the different parameters extracted from the cooccurrence matrix are calculated, color information and color distances are taken into account. Instead of calculating differences such as |i−j|, where i and j are the color indexes of the quantized image, color differences, ΔE, between a color with index i, $C_{i}$ and a color with index j, $C_{j}$, are calculated (ΔE is defined in Equations (1) and (6) for L*a*b* and CAM16-UCS color spaces, respectively). Thus, the main parameters extracted from this cooccurrence matrix are calculated as follows:

Homogeneity

$H=\overset{20,20}{\underset{i,j}{\sum }}\frac{P_{ij}}{1+\left(\Delta E(C_{i},C_{j}\right)\;{)}^{2}}$

Mean

$\mu =\overset{20,20}{\underset{i,j}{\sum }}C_{i}P_{ij}$

Variance

$\sigma^{2}=\overset{20,20}{\underset{i,j}{\sum }}\left(\Delta E(C_{i},\mu_{i}\right)\;{)}^{2}P_{ij}$

Correlation

$\rho =\overset{20,20}{\underset{i,j}{\sum }}\frac{\Delta E(C_{i},\mu_{i})\Delta E(C_{j},\mu_{j})}{\sqrt{\sigma_{i}^{2}\sigma_{j}^{2}}}\;P_{ij}$

Entropy

$S=\overset{20,20}{\underset{i,j}{\sum }}-P_{ij}\ln\left(P_{ij}\right)$



Homogeneity measures the spatial closeness of the distribution of elements in the CCM to the diagonal. The CCM mean coincides with the image mean. CCM variance is a measure of the contrast between a pixel and its neighbors. Correlation is a measurement of how a pixel correlates to its neighbor across the entire image. Entropy is a measure of variability and it is 0 for a constant image.

In this sense, perceptual color differences are taken into account, and a new color cooccurrence matrix has been defined. Cooccurrence matrix parameters are color perception-adapted because, in all these parameters, perceptually uniform color differences, ΔE, are applied.

Texture information is then employed in a conventional machine learning module that is combined with the deep learning architecture. Two different configurations of this machine learning module are tested:

(1) The inputs to the module are the GLCM parameters calculated from the L* channel;
(2) The inputs to the module are the new CCM parameters.

Both architectures are described in Section 2.5, Test 3.

### 2.5. Classification

**Figure 5:**
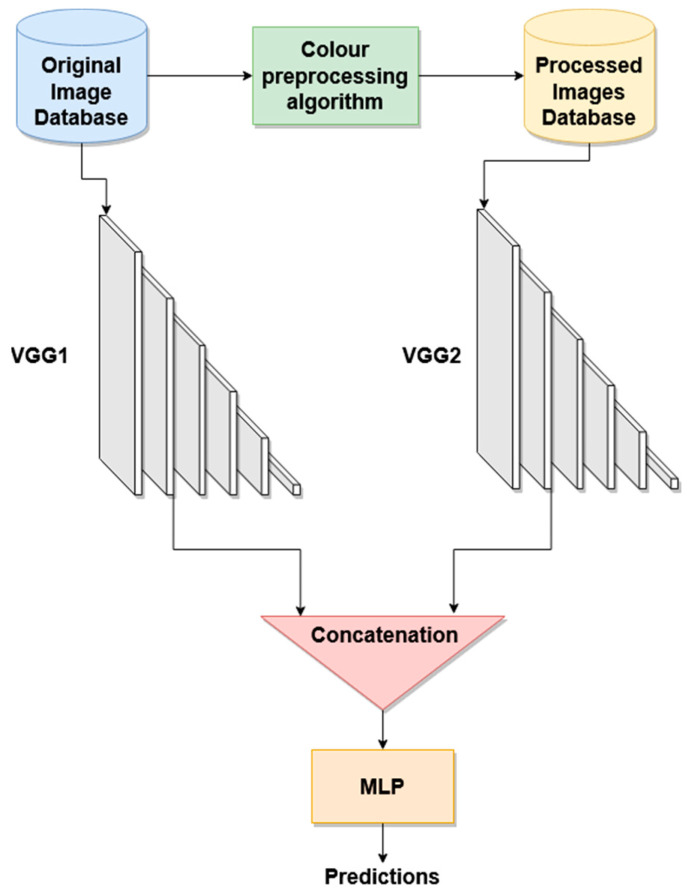
Dual classification. Original *RGB* and color-quantized images are used as inputs to the VGG16 CNNs, and concatenated to enter in a MLP classifier.

**Figure 6:**
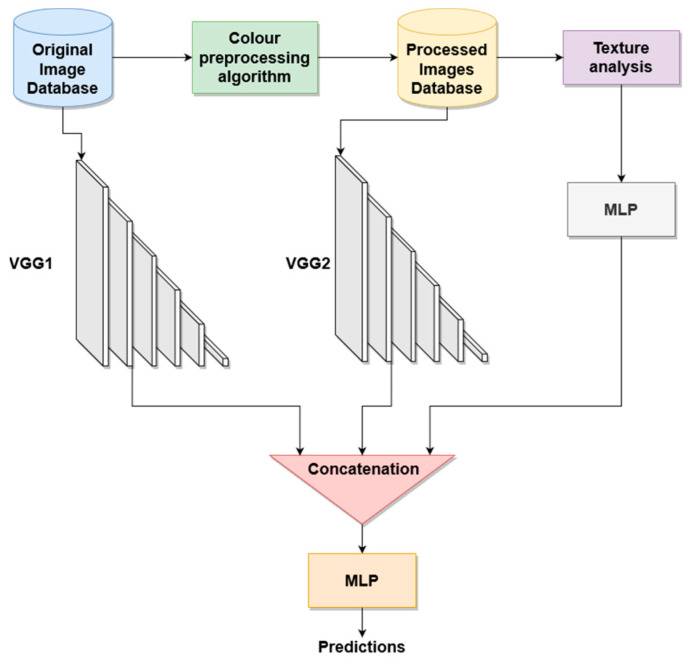
Triple classification. Original *RGB*, color-quantized images and texture descriptors are used as inputs to the MLP classifier.

For the classification into the different BCC patterns, three different architectures are proposed. These architectures employ the convolutional layers of a deep convolutional network, fully connected layers optimized for this application and a multilayer perceptron to concatenate the outputs of the different blocks, as shown in Figure 5 and Figure 6.

To choose the convolutional layers, different neural networks were tested: Inception V3, Vgg16, ConvNet and EfficientNetB0. VGG16 obtained the best classification, so the VGG16 neural network has been chosen [58]. Specifically, the first convolutional layers have been pre-trained with the ImageNet dataset [59] and these layers have been employed as building blocks in the different architectures proposed.

Subsequently, new fully connected layers (FCL) designed specifically for this problem have been added. These FCLs, along with the last convolutional layers, were fine-tuned for this specific problem. The number of neurons of these FCLs has been optimized to obtain the best classification results.

#### 2.5.1. Architecture 1: Classification with Original *RGB* Images

In the first scenario, the inputs to the CNN were the original *RGB* images (256 × 256 pixel images, where one or more patterns are present). The images were weakly annotated by specialists. The CNN in this architecture consists of the convolutional layers of a VGG16 and one fully connected layer.

#### 2.5.2. Architecture 2: Classification with Original *RGB* Images along with Color-Quantized Images in Different Color Spaces (Dual Classification)

In the dual configuration, the original *RGB* image and the color-quantized one are the inputs to the convolutional layers of two VGG16 CNNs, respectively, followed by one fully connected layer, as shown in Figure 5. The outputs of the two CNNs are then concatenated and feed a multilayer perceptron (MLP) classifier.

#### 2.5.3. Architecture 3: Classification with Original *RGB* Images, Color-Quantized Images in Different Color Spaces and Texture Features (Triple Classification)

In an attempt to overcome the problems of a small database, we introduce a conventional machine learning module, where texture features are extracted. The classification architecture is described in Figure 6. The texture features used in this test are described in Section 2.4. Thus, two tests have been performed. In the first triple configuration test, the GLCM texture features were calculated. In the second triple configuration test, the CCM texture features were computed. Before concatenating them with the dual classification module, the texture descriptors pass through an MLP classifier. The architecture of this MLP has been optimized by classifying the training images exclusively with these texture parameters.

As multiple BCC dermoscopic features can be found in the same image, the three proposed architectures have been trained as follows. A common architecture with 7 neurons in the last layer, corresponding to the 7 BCC patterns given in Table 1, was utilized. Each neuron in this last layer provides a probability, which is thresholded to provide the final classification. The area under the curve (AUC) is determined by varying the threshold. This network was trained with binary cross-entropy loss to take into account that multiple classes can be assigned to the same image.

#### 2.5.4. Classification of BCC and Non-BCC

According to Peris et al. and Menzies et al. [2,25,60], dermoscopic criteria for BCC are the absence of brown reticular lines (pigment network) together with the presence of any of the following 6 dermoscopic features: telangiectasia, ulceration, blue-gray ovoid nests and globules, leaf-like areas, spoke-wheel areas and clods.

Thus, in the proposed algorithm, once these 7 dermoscopic features have been identified, all the lesions are classified into BCC or non-BCC following this clinical criterion.

### 2.6. Description of the Hardware and Software Used

The proposed method has been implemented in a computer with an Intel Core i9 with a Nvidia Titan RTX 24GB GPU.

The code corresponding to the algorithms described in this paper can be found at https://github.com/ManuelL4z0/dermaBCC (accessed on 7 July 2022). The software has been developed with Python 3.8.5, the Anaconda 4.9.2 environment and Spyder 4.1.5.

## 3. Results

**Figure 7:**
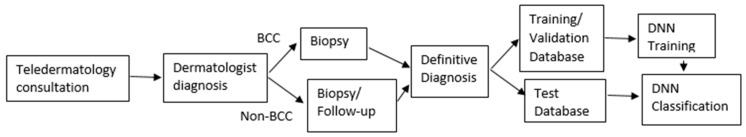
Flow diagram followed by the participants in the four tests analyzed in this work.

A summary of the flow diagram followed by the participants in the tests conducted in this work is presented in Figure 7.

The four tests carried out are as follows:

*Test* 1: Architecture 1 is used with the original *RGB* images as inputs. The classification’s objective is to classify the dermoscopic patterns present in the lesions.

*Test* 2: Architecture 2 is used with the original *RGB* images and the color-quantified images as inputs. The classification’s objective is to classify the dermoscopic patterns present in the lesions.

*Test* 3: Architecture 3 is used, where the inputs are the original *RGB* images, the color-quantified images and the texture features extracted from the cooccurrence matrix as inputs. The classification’s objective is to classify the dermoscopic patterns present in the lesions.

*Test* 4: Classification of the images into BCC and non-BCC lesions. If one or more BCC patterns are detected in the images using one of the above tests, the image is classified as BCC. If a pigment network pattern or no pattern is detected using one of the above tests, the image is classified as non-BCC.

For the training of the CNNs, a batch size of 32 was used. The learning rate was adaptive and the optimization algorithm was AdamGrad. The loss function was the binary cross-entropy.

Table 2 provides an analysis of how the information about the distribution of colors in the image influences the final classification. To this aim, in Test 1, whose results are shown in the first row, the network was fed exclusively with the original images. The rest of the rows show the classification results when, along with the original image, the quantized image with the distribution of the main colors was introduced as input to the neural network. Three different color spaces were analyzed.

As shown in Table 2, the configuration of Test 1 attained poor results. In this regard, sensitivity of 0.48 is achieved for the spoke-wheel dermoscopic features and specificity under 0.7 is attained for three dermoscopic features.

When the configuration of Test 1 is compared to Test 2, the best classification results are attained with the dual network when it is fed with the original image plus the CIECAM16-quantized image, which improves the AUC obtained with CIELAB quantization for all the BCC dermoscopic features except for spoke-wheel. The poor results obtained for spoke-wheel and multi-globules, with AUC under 0.8, can be explained by the limited training data available for these BCC dermoscopic features. In the same way, the sensitivity obtained for these dermoscopic features is poor, which can be explained by the class imbalance present in the database.

As described in the Materials and Methods section, due to the small number of training images, it was considered that the classification results could be improved if texture hand-crafted features were introduced to the classifier along with the image. In the first few rows of Table 3, results obtained when the handcrafted features introduced to the classifier were GLCM parameters extracted from the lightness component, L*, are shown. In the last few rows of Table 3, the handcrafted features introduced to the network are the proposed CCM features.

As can be observed in Table 3, the introduction of GLCM parameters to the classifier improves slightly the classification results. Again, when CIECAM16 quantization is employed, the results are slightly better.

Another important observation is that CCM parameters improve the classification. First, the AUC is over 0.8 for all BCC feature classifications when CCM features are applied. Secondly, the sensitivity, specificity and AUC are higher than those obtained with GLCM parameters.

**Figure 8:**
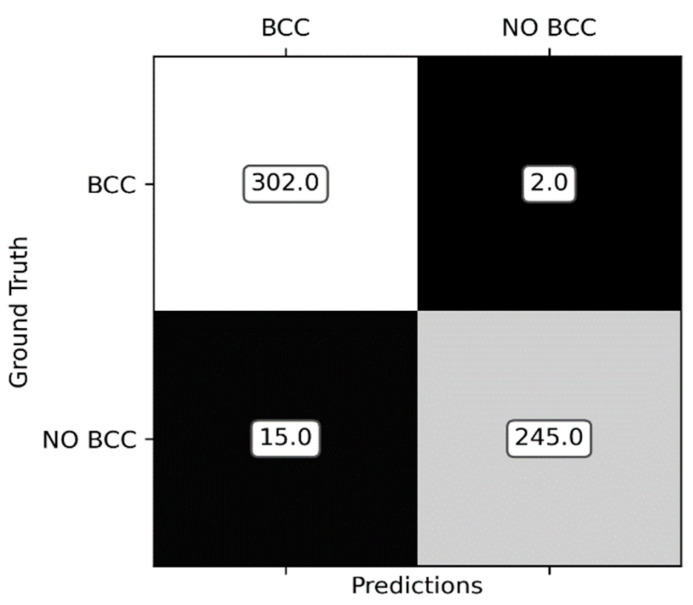
Confusion matrix of the classification into BCC versus non-BCC.

Finally, in Table 4, results when classifying all the lesions into BCC versus non-BCC are shown, wherein the BCC dermoscopic features have been estimated with the triple network and CCM features. As can be observed, the best results are attained when color differences are estimated in CIECAM16, achieving sensitivity of 0.9934. Figure 8 shows the confusion matrix of the classification into BCC versus non-BCC.

**Figure 9:**
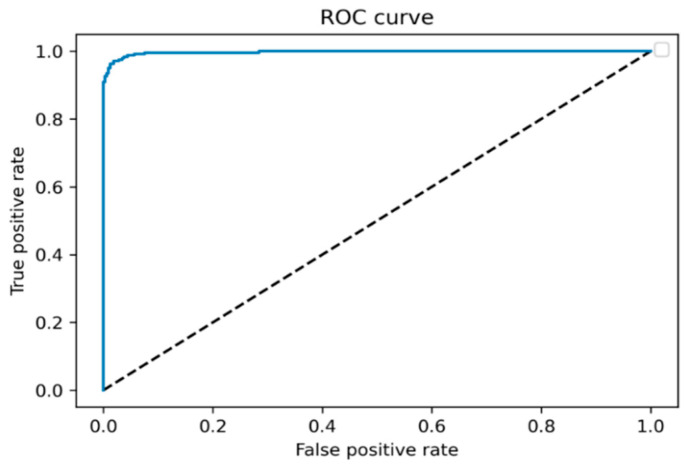
ROC curve when classifying BCC versus non-BCC.

In addition, the ROC curve was calculated when CIECAM16 was used for color quantization, which is shown in Figure 9. The AUC was also computed and was equal to 0.997.

## 4. Discussion

This work is focused on the detection of specific patterns belonging to BCC. Given the presence or absence of these patterns, dermatologists diagnose BCC. In this sense, this work tries to emulate the dermatologist’s assessment in order to detect BCC.

To the best of our knowledge, no other work in the literature detects all the BCC dermoscopic clinical features that clinicians use to diagnose BCC lesions. There are some works focusing on the detection of one or several dermoscopic criteria [29,61], but none of them use them to give an explainable classification into BCC and non-BCC.

Most of the works found in the literature classify skin lesions into several diseases, including BCC as one of these diseases. Usually, they employ conventional machine learning algorithms [62,63] or deep learning-based methods [10,64]. These methods do not provide an explanation of this classification, which could assist the physician in their assessment.

Color is one of the main attributes on which physicians base their diagnosis of skin lesions. Different authors have employed color features to classify skin lesions. We demonstrate that the introduction of the spatial distribution of the main colors present in the image, i.e., a quantized image, improves the classification results compared to the results using only the original images (AUC = 0.85 vs. 0.83). It should be noted that this color quantization is perceptual, in the sense that colors that are perceptually similar have been grouped within the same color cluster.

In a recent paper, in a prospective study Sies et al. compared a conventional machine learning method with a CNN to classify skin lesions [65]. They concluded that the CNN method outperformed methods based on handcrafted features. However, in this paper, we demonstrate that the addition of handcrafted features to a CNN architecture can improve the results, especially when the number of training samples is small. Specifically, an AUC equal to 0.92 has been achieved, versus an AUC equal to 0.85 when the classifier is a CNN architecture whose input is exclusively the original image.

Different authors have demonstrated that color–texture features outperform texture features [56]. Thus, the investigation of new color–texture features is of great interest in color image classification. In this paper, new color–texture features based on a color cooccurrence matrix resulting from the perceptually quantized color image are proposed. The improvement of this proposed color cooccurrence matrix (CCM) versus the gray-level cooccurrence matrix (GLCM), even when color information has been previously introduced to the network, has been demonstrated (AUC = 0.92 vs. 0.88).

Finally, the classification into BCC and non-BCC based on the patterns found in the lesions has been performed. The high classification rate obtained (SE = 0.99) demonstrates that a classification based on the clinical diagnostic criteria can attain very good results. In addition, this classifier provides an explainable classification to the clinician.

In this paper, the STARD 2015 updated list of essential items for reporting diagnostic accuracy in studies has been followed [66].

### 4.1. Future Plans

The proposed work shows promising results. However, the small database has limited the quality of the results. Thus, we are working on a new version of the annotation software tool with the ability of creating segmentation masks, which will facilitate and accelerate the annotation task and, consequently, will allow the database to increased. This tool will provide not only a weakly annotated database, but also the location of the BCC feature within the image. 

If the database keeps improving and growing, the methods shown should also increase in their classification capability, making easier and more profitable the integration with the current teledermatology system. If it were to become operative, as the amount of BCC cases processed at Hospital Universitario Virgen Macarena is around 300 in a month, the algorithm could become more and more efficient and generalized as time goes by.

A prospective study is intended to be carried out. The developed tool will be installed in the computers of the hospital. The tool will be employed to evaluate all the images received at the hospital by the teledermatology team, and the classification results will be compared with the dermatologist’s diagnosis and with the clinical judgement of the general practitioner who acquires the dermoscopic image at the primary health center. At the Virgen Macarena Hospital and its healthcare area, involved in this project, there are 315 dermatology teleconsultations in a month on average, and 100 of these teleconsultations result in a BCC diagnosis. This can serve as an estimation of the amount of data that could be available for a prospective study. 

### 4.2. Strength and Limitation

In summary, the main strength of this study is that it provides a classification accompanied by an explanation, which includes information about the BCC dermoscopic features found in the lesion. Physicians prefer an explained classification rather than a binary classification.

On the other hand, the main limitation is that all the results have been obtained with images collected retrospectively. Most of the results published in the literature are based on retrospective studies. Thus, although the accuracy of computer-aided diagnosis for skin lesion detection is comparable to that of experts, the real-world applicability of these systems is unknown.

## 5. Conclusions

In this paper, we propose a DNN architecture to detect the dermoscopic patterns that clinicians employ to discriminate between BCC and non-BCC skin lesions. In this architecture, together with the original image, a quantized color image and color–texture handcrafted features are introduced as inputs to the network, improving the classification results. The quantized color images have been quantized according to perceived color differences obtained from a uniform color space derived from CIECAM16 [53]. Color–texture handcrafted features are new, perceptually inspired color features derived from a color cooccurrence matrix.

The improvement achieved with this methodology over a DNN fed only with original *RGB* images is the following: the specificity parameter has increased from 0.75 to 0.82, the sensitivity parameter has increased from 0.77 to 0.90, and the AUC has increased from 0.83 to 0.92.

Finally, when the lesion is classified into BCC or non-BCC based on the dermoscopic features found, sensitivity of 0.9934 is achieved.

Thus, this classification can provide an accurate and explainable classification to the physician.

## Figures and Tables

**Table 1:** Number of images for each pattern in the database.

	Number of Occurrences
Pigment Network	614
Ulceration	352
Blue-Gray Ovoid Nests	338
Multiple B/G Globules	150
Maple Leaf	177
Spoke-Wheel	64
Arborizing Telangiectasia	526

**Table 2:** Evaluation results for Test 1 and Test 2 classifications. SPEC: specificity; SENS: sensitivity; AUC: area under the curve.

		SPEC	SENS	AUC
**Original RGB**	Pigment network	0.89	0.91	0.95
	Ulceration	0.74	0.87	0.87
	Ovoid nest	0.61	0.83	0.77
	Multiple globules	0.69	0.66	0.74
	Maple leaf	0.69	0.76	0.79
	Spoke-wheel	0.90	0.48	0.83
	A. telangiectasia	0.71	0.88	0.88
	**Average**	**0.75**	**0.77**	**0.83**
**Dual** **original + *RGB* quantization**	Pigment network	0.95	0.97	0.99
	Ulceration	0.82	0.70	0.85
	Ovoid nest	0.70	0.68	0.77
	Multiple globules	0.74	0.63	0.75
	Maple leaf	0.86	0.72	0.86
	Spoke-wheel	0.95	0.36	0.88
	A. telangiectasia	0.77	0.79	0.85
	**Average**	**0.83**	**0.69**	**0.85**
**Dual** **original + CIELAB quantization**	Pigment network	0.94	0.95	0.98
	Ulceration	0.81	0.86	0.91
	Ovoid nest	0.65	0.73	0.76
	Multiple globules	0.60	0.68	0.71
	Maple leaf	0.78	0.72	0.82
	Spoke-wheel	0.84	0.73	0.85
	A. telangiectasia	0.73	0.88	0.87
	**Average**	**0.77**	**0.79**	**0.84**
**Dual original + CIECAM16 quantization**	Pigment network	0.95	0.96	0.98
	Ulceration	0.86	0.79	0.92
	Ovoid nest	0.71	0.76	0.80
	Multiple globules	0.65	0.72	0.77
	Maple leaf	0.69	0.78	0.83
	Spoke-wheel	0.80	0.61	0.78
	A. telangiectasia	0.75	0.87	0.88
	**Average**	**0.78**	**0.78**	**0.85**

**Table 3:** Evaluation results for Test 3 classification SPEC: specificity; SENS: sensitivity; AUC: area under the curve.

		SPEC	SENS	AUC
**Triple** **original + CIELAB** Quantization + GLCM L*	Pigment netw.	0.92	0.99	0.99
	Ulceration	0.80	0.82	0.90
	Ovoid nest	0.71	0.68	0.80
	Multiple globules	0.78	0.77	0.86
	Maple leaf	0.77	0.69	0.81
	Spoke-wheel	0.72	0.86	0.89
	A. telangiectasia	0.72	0.85	0.87
	**Average**	**0.77**	**0.81**	**0.87**
**Triple** **original + CIECAM16** quantization + GLCM L*	Pigment netw.	0.97	0.97	0.98
	Ulceration	0.76	0.81	0.87
	Ovoid nest	0.60	0.88	0.79
	Multiple globules	0.78	0.79	0.86
	Maple leaf	0.80	0.77	0.85
	Spoke-wheel	0.79	0.71	0.89
	A. telangiectasia	0.73	0.79	0.89
	**Average**	**0.78**	**0.82**	**0.88**
**Triple** **original + CIELAB quantization+ CIELAB CCM**	Pigment netw.	0.98	0.97	0.99
	Ulceration	0.82	0.75	0.89
	Ovoid nest	0.74	0.84	0.86
	Multiple globules	0.78	0.68	0.80
	Maple leaf	0.78	0.68	0.85
	Spoke-wheel	0.89	0.97	0.96
	A. telangiectasia	0.76	0.87	0.91
	**Average**	**0.82**	**0.82**	**0.89**
**Triple** **Original + CIECAM16 quantization+ CIECAM 16 CCM**	Pigment netw.	0.98	0.97	0.99
	Ulceration	0.86	0.92	0.94
	Ovoid nest	0.85	0.83	0.91
	Multiple globules	0.79	0.87	0.89
	Maple leaf	0.72	0.82	0.82
	Spoke-wheel	0.87	0.93	0.96
	A. telangiectasia	0.68	0.99	0.91
	**Average**	**0.82**	**0.90**	**0.92**

**Table 4:** Results of the classification into BCC versus non-BCC. ACC: accuracy; PPV: positive predictive value; SPEC: specificity; SENS: sensitivity.

	ACC	PPV	SPEC	SENS
**CIELAB**	0.9685	0.9789	0.9703	0.9673
**CIECAM16**	0.9699	0.9527	0.9423	0.9934

## Data Availability

The data presented in this study are available on request from the corresponding author. The data are not publicly available due to ethical restrictions.
